# Research and intervention priorities for mental health of people living with chronic disease(s) in the midst of the COVID-19 pandemic in low resource settings: A commentary

**DOI:** 10.1016/j.amsu.2020.07.051

**Published:** 2020-08-07

**Authors:** Tsegaye Melaku, Desta Assefa, Bodena Bayisa, Negase Legese

**Affiliations:** a School of Pharmacy, Institute of Health, Jimma University, Ethiopia; b School of Medicine, Institute of Health, Jimma University, Ethiopia; c Jimma University Medical Center, Jimma, Ethiopia

In late December 2019, Wuhan city, the capital of Hubei province in China, announced an outbreak of respiratory infection of unknown cause. Following this announcement, the World Health Organization declared a Public Health Emergency of International Concern (PHEIC) on January 30 from the 2019 coronavirus disease (COVID-19) [1]. Since then, the world has been shocked by the wave of COVID-19 caused by severe acute respiratory syndrome coronavirus-2 (SARS-CoV-2). Following the outbreak, the psychological issues which accompany this pandemic have rapidly compounded its public health burden [2]. The public health impact of the pandemic in low-income countries, particularly in Africa, that already faces struggling health care systems and a scarcity of skilled health workers, is of grave concern [3], especially for those people living with communicable and non-communicable chronic illnesses. The COVID-19 pandemic obliged many of the people to stay at home and doing less in terms of social interactions and exercise. Across the globe, as the COVID-19 pandemic has overwhelmed the health care system, people living with chronic illnesses have been forced to postpone much of their clinical care, which can be critical to keeping their most troublesome symptoms at bay [4]. For people with multiple chronic conditions, or those who have had past issues managing an aspect of their condition, hospital or laboratory closures could prove particularly problematic, especially if they have been relying on regular check-ins with practitioners or regular laboratory tests to keep their conditions in check.

Following the global outbreak of the COVID-19 [5], different quarantine policies and COVID-19 prevention strategies have been implemented in different countries to control the epidemic in time. In this circumstance, patients with chronic diseases, such as heart failure, hypertension, diabetes, pulmonary disease, and so on, would suffer from emotional disturbance, anxiety, anger, confusion, and stigma [6, 7] because of sudden separation from loved ones, shortage of living supplies, the loss of freedom, and uncertainty over disease status. Also, some patients have been confronted with difficulties in routine medical treatments due to delayed transportation and shortages of medicines and human resources in hospitals [8]. Even several old patients with chronic diseases dare not go to the hospital. All these situations increase the possibility of relapse or even death. For example, from the experience of the 2002–2004 SARS outbreaks, chronic-care hospitalizations for diabetes plummeted during the first phase of crisis but skyrocketed afterward. If the problem related to the treatment of the outcome of chronic illness is not managed on time a similar problem will crop up as a result of the COVID-19 pandemic. Besides, social distancing policies that will not consider the already precarious existence of many older people living with chronic diseases particularly those living alone or dependent on others for care and support will bear the psychological and physical brunt of COVID-19. In low resource settings, especially in Africa, older people may face barriers to obtaining food and other essential supplies if quarantine conditions become more widespread. In Africa, because of the coronavirus pandemic is getting worse, the authorities have decided to declare a state of emergency, which include restriction of unnecessary mobility, mass gatherings, reduction of transport services, etc, which will impose both physical and psychological impact on the non-COVID-19 patients seeking for health care, specifically patient on long term care.

Emerging research outputs assessing the mental health associated with COVID-19 has identified a heightened prevalence of moderate-to-severe self-reported different phenotypes of mental health symptomatology among the general public [9]. However, further research that investigates high-risk populations to understanding the disruption of routines and clinical care of chronic illness as a result of COVID-19, and its associated psychological and physical impacts are highly required in low resource settings.

These preliminary findings from high resource settings highlight the multiple factors contributing to aggravating underlying mental health problems or the occurrence of incident mental health problems among people living with a chronic illness during this pandemic. However, there remains much to be learned about the psychological and physical impacts facing these high-risk groups and what can be done to reduce their negative effects in the low resource settings, where a multitude of health, economic, social, and political problems are looming together [10]. A timely call to action for further research examining the impact of COVID-19 on the mental health of people living with a chronic illness is suggested. Specifically, priorities should include novel intervention for enabling disease state control, promotion of easy access to prescription refill, increase in adherence to regimen and on time referral or consultation in supporting the physical and psychological care during the COVID-19 outbreak. New evidence may help to inform individually tailored patient-centered support programs and mitigate the long-term negative implications for patient life and mental health. New innovative clinical cares enabling solutions such as telehealth interventions (i.e. mobile health, telemedicine, telepharmacy) are very critical for real-time patient management both for primary disease conditions and mental health problems.

## Ethical approval

Not applicable.

## Source of funding

The author(s) received no financial support for the research, authorship, and/or publication of this article.

## Author contribution

Tsegaye M and Desta A drafted the commentary. Tsegaye M, Bodena B and Negase L provided critical input and edits. All authors read and approved the final manuscript.

## Registration of research studies

1. Name of the registry: Not applicable
2. Unique Identifying number or registration ID: Not applicable
3. Hyperlink to your specific registration (must be publicly accessible and will be checked): Not applicable

## Guarantor

Tsegaye Melaku.

## Consent

Not applicable.

## Availability of data and materials

- Not applicable.

## Provenance and peer review

Not commissioned, externally peer reviewed.

## Declaration of competing interest

No competing interests exist.
